# Children Immunization App (CImA) Among Syrian Refugees in Zaatari Camp, Jordan: Protocol for a Cluster Randomized Controlled Pilot Trial Intervention Study 

**DOI:** 10.2196/13557

**Published:** 2019-10-07

**Authors:** Yousef S Khader, Lucie Laflamme, Daniela Schmid, Soha El-Halabi, Mohammad Abu Khdair, Mathilde Sengoelge, Salla Atkins, Manal Tahtamouni, Tarik Derrough, Ziad El-Khatib

**Affiliations:** 1 Department of Community Medicine, Public Health and Family Medicine Faculty of Medicine Jordan University of Science & Technology Irbid Jordan; 2 Department of Public Health Sciences Karolinska Institutet Stockholm Sweden; 3 Department for Infectious Disease Epidemiology Austrian Agency for Health and Food Safety (AGES) Vienna Austria; 4 Department of Learning, Informatics, Management and Education Karolinska Institutet Stockholm Sweden; 5 National vaccination program Ministry of Health Amman Jordan; 6 New Social Research and Faculty of Social Sciences Tampere University Tampere Finland; 7 Health and Nutrition UNICEF Amman Jordan; 8 European Centre for Disease Prevention and Control Stockholm Sweden; 9 Department of Community Medicine, Public Health and Family Medicine Faculty of Medicine Jordan University of Science & Technology Irbid Jordan

**Keywords:** mHealth, refugees, vaccines, Jordan, Syria

## Abstract

**Background:**

There are up to 19.4 million children who are still unvaccinated and face unnecessary deaths, especially among refugees. However, growing access to smartphones, among refugees, can be a leading factor to improve vaccination rates.

**Objective:**

This study aims to determine whether a smartphone app can improve the vaccination uptake among refugees and determine the app’s effectiveness in improving the documentation of vaccination records.

**Methods:**

We developed and planned to test an app through a cluster randomized trial that will be carried out at the Zaatari refugee camp in Jordan. The study will be open to all parents who carry Android smartphones, have at least one child, and agree to participate in the study. The parents will be recruited to the study by trained volunteers at the vaccination sites around the Zaatari camp. Inclusion criteria will be the following: having at least one child of 0 to 5 years, being a local resident of the camp, and having an Android smartphone.

**Results:**

The intervention includes an app that will allow storing Jordanian vaccination records, per child, on the parents’ smartphones in Arabic and English (in an interchangeable fashion). Every record will have a set of automated reminders before the appointment of each child. The app will summarize immunization records in form of due, taken, or overdue appointments, labeled in orange, green, and red, respectively. Baseline will include the collection of our primary and secondary outcomes that are needed for the pre and postdata measurements. This includes social demographic data, any previous vaccination history, and electronic health literacy. Participants, in both study arms, will be monitored for their follow-up visits to the clinic for vaccination doses. For the study outcome measures, we will measure any differences in the uptake of vaccinations. The secondary outcome is to analyze the effect of the children immunization app on visits for follow-up doses.

**Conclusions:**

Owing to the limited evidence of effective interventions for childhood vaccination among refugees, research in this area is greatly needed. The project will have a significant impact on the health of refugees and the public health system. In Jordan and the Middle East, the vaccination level is low. Given the influx of refugees from the area, it is crucial to ensure a high vaccination level among the children.

**International Registered Report Identifier (IRRID):**

PRR1-10.2196/13557

## Introduction

### Background

Child immunization is a key component of the World Health Organization and the United Nations Children’s Fund (UNICEF)’s child survival intervention package [1]. Immunizing children is an essential strategy in the primary health care services, and it is a crucial public health objective. However, internationally, there are up to 19.4 million children who are still unvaccinated and face unnecessary deaths [2], especially among refugees [3]. Owing to this, refugees from conflict-torn countries have a high risk of acquiring Vaccine Preventable Diseases (VPDs) [4], especially as half of the refugees are below 18 years [5]. In conflict-torn countries, such as Syria, the immunization systems and follow-up, along with information systems, have been weakened, and herd immunity has been compromised. Before the crisis, Syria had greater than 80% of immunization coverage [6], which reduced to below 50% during the period 2011 to 2015. The total number of measles cases also increased from 13 to 740 in late 2011 and early 2013 (approximately an increase of 57-fold in less than 12 months) [2]. Since 2011, over 3 million children, aged 0 to 5 years, have fled out of Syria, to the Middle East and Europe [7]. These children have been exposed to several risky situations on the way to their new hosting countries, including passing or living in countries with low vaccination coverage [6]. In 2017, Roberton et al reported that the vaccination rate among Syrian children was 25% and 13% in Jordan and Lebanon, respectively [8]. Therefore, they are at a high risk of acquiring VPDs. This causes a heightened risk of unnecessary outbreaks in host countries, particularly among individuals who have not been vaccinated at all or completed vaccination schedules, whether refugee or from the host population [9]. The estimated cost of hospitalization may be up to US $25,000 per case [10]. The influx of children without clear immunization records therefore creates a challenge for health providers to maintain herd immunity and vaccinate unvaccinated children [5,9,11,12]. At the same time, the vaccination rate in the hosting country is considered low, for example, in Jordan, the vaccination rate among the general population is estimated to be less than 50% [13]. Given the risk to unvaccinated children, both in the refugee populations and the general population, it is paramount that support is given to health professionals to monitor and increase the rate of vaccination among refugee children. The challenge for monitoring vaccinations among refugees is compounded by the use of the yellow vaccination cards, which are easily lost or not brought to medical consultations. The field of mobile Health (mHealth) could be used as an alternative for paper-based vaccination records, and a smartphone app could present advantages to empower parents by informing them of vaccination schedules and dates and allowing them to monitor vaccination coverage on their own. There is growing evidence on the effectiveness of smartphone technologies in supporting health care services [14], though there remain gaps in the evidence of effectiveness. Smartphones provide a novel approach to solve problems with data registration, transmission, and storage [15-18]. Currently, there are over 5.2 billion users, in low- and middle-income countries [19]. The number of purchased cell phones is expected to exceed the total number of people on earth [20]. The emergence and spread of smartphones have provided a novel approach toward improving access to health care services [17]. The advancement of technology has provided tools that can empower users [21], patients, and other populations at risk. In general, mHealth can be useful among the population and among vulnerable groups, such as refugees [22]. Refugees and other vulnerable populations use smartphones as a survival kit, to connect with their social networks and to search for information about their host countries [10]. Anecdotally, women are the main caregivers for their children, and they are active users of smartphone technologies, so they can stay in touch with their social network and seek information regarding the integration process in the hosting countries [23]. It is therefore possible to use technological innovations, such as apps, to develop appropriate tools to support both women and men refugees, as well as health care professionals involved in the delivery of urgent health services. Health technology innovation is playing a crucial role in helping refugees, in various contexts around the world, including low-, middle-, and high-income countries [24].

However, there remains a lack of well-conducted evaluations of mHealth interventions among refugee populations, particularly with respect to maternal and child health. According to our knowledge, no study has used an app to support refugees’ population in recording their vaccination records and provide them an automated reminder for the vaccination visits. The purpose of this study is to develop and evaluate an mHealth intervention that helps Syrian refugees to store their immunization and health records to facilitate health information sharing at the Zaatari camp in Jordan.

### Objectives

The objectives are as follows:

- To implement an integrated app intervention in collaboration with UNICEF, Ministry of Health, United Nations High Commissioner for Refugees (UNHCR), and local health service delivery partners
- To analyze the uptake of vaccination coverage (primary outcome)
- To analyze the effect of the smartphone app intervention on visits for the follow-up doses (secondary outcome)
- To conduct a thorough process evaluation of the intervention implementation

## Methods

### Study Setting

The study will be conducted at the Zaatari camp in Jordan (5.3 km^2^), which is considered one of the largest hosting camps for refugees in Jordan and the Middle East. The camp was first opened in 2012, to host the Syrian refugees fleeing the Syrian civil war. The camp population is estimated to be hosting 80,000 refugees (approximately 15,000 persons per km^2^), where approximately 20% (n=16,000) of them are under 5 years.

### Study Design

This will be a cluster randomized controlled trial to evaluate the effectiveness of using an app to record the vaccination schedule, including reminders for parents, on increasing immunization coverage of Syrian children at the Zaatari refugee camp in Jordan. In March 2019, the study was announced through posters in Arabic, in the clinics. Clinicians and social workers will also inform the residents of the camps about the study. Parents interested in joining the study will be fully briefed about the study, and an informed consent form will be signed. After completing the consent form, participants (1) included in the app intervention arm (ie, children immunization app [CImA] recipients) will receive the usual care, access to the CImA app, and instructions on how to use it, in addition to updating their children vaccination card, or (2) under the usual care (control arm) of the trial, where they will receive the usual information on the benefits of vaccination, the child’s yellow vaccination card will be updated, in addition to the list of appointments for the future vaccines. The vaccination sites will be enrolled on the basis of convenience, because of the security situation in the refugee camp.

### Sample Size Calculation

The sample size was calculated using G*Power 3.1.9.2 for testing the effect of the intervention by McNemar test. For 1-tailed hypothesis, at a power of 80% and level of significance of 0.05, a sample of 374 children is needed to detect a clinically important change in the proportion of children who will come back for their follow-up doses after introducing the intervention (ie, odds ratio of 1.5) [25]. After adjusting to potential proportion of lost to follow-up, the estimated sample size becomes 535. The analysis will be performed using Stata Statistical Software: Release 14 (College Station, TX: StataCorp LP.).

### Children Immunization App Description

The CImA app includes 5 layers: (1) health promotion messages for the benefits of vaccination, which show up on the main page; (2) storing the post of vaccination for each child, according to the vaccination schedule of the Jordan Ministry of Health, on the parents’ smartphones; (3) displaying the vaccination schedule, for each child, using green, orange, and green colors, depending on vaccination status if it was received, due or overdue, respectively; (4) appointment reminder will be displayed on the users’ phones at 4 different time points before the vaccination schedule (1 week, 3 days, and 1 day and the morning of the appointment). Thereafter, the user will receive 2 notifications in the coming days of the scheduled vaccine in case of missing the appointment.

Participants will be able to download the CImA app, at no cost, on their personal devices (Android only), with the help of the study staff (we will make the link invisible to public access during the study recruitment period); and (5) the stored information is in Arabic and English (in an interchangeable fashion).

### Intervention

**Figure 1:**
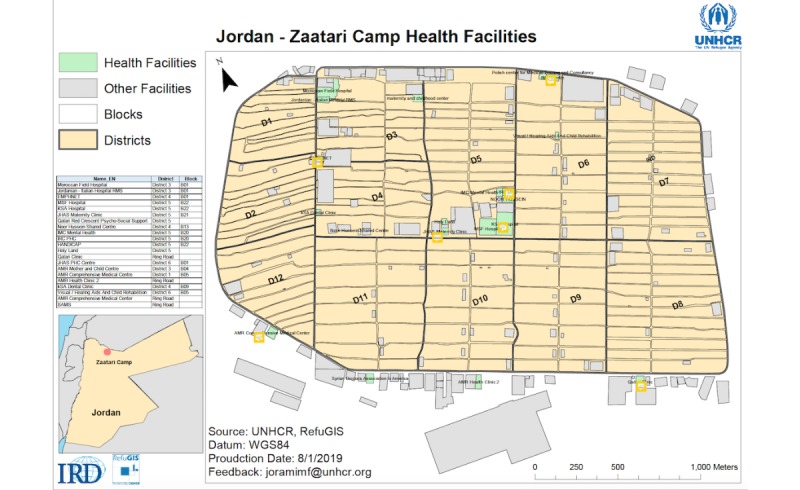
The distribution of districts at the Zaatari camp and the location of the health facilities providing vaccinations. A complete map for the Zaatari camp can be found at https://data2.unhcr.org/en/documents/download/55994

The parents will be recruited to the study by trained volunteers at the 7 local clinics, providing vaccinations, around the Zaatari camp (Figure 1). Inclusion criteria will be the following: having at least one child of 0 to 5 years, being a local resident of the camp, and having an Android smartphone that can allow CImA app installation.

The intervention includes an app that will (1) allow storing Jordanian vaccination records (Table 1), per child, on the parents’ smartphones, in Arabic and English (in an interchangeable fashion), (2) have a set of automated reminders before the appointment of each child, and (3) in addition, summarize immunization records in the form of *due*, *taken*, or *overdue* appointments, labeled in orange, green, and red, respectively.

**Table 1:** Summary of the national vaccination schedule of the Jordan Ministry of Health.

Age	Recommended vaccines
1st contact	BCG^a^
2 months	DTaP-Hib-IPV + HBV^b^; RV^c^ 1st
3 months	DTaP-Hib-IPV + HBV; RV 2nd; OPV^d^
4 months	DTaP-Hib-IPV + HBV; RV 3rd; OPV
9 months	Measles; OPV
12 months	MMR1^e^
18 months	DTP^f^; MMR2; OPV
6 years (first grade)	Td^g^; Check for MMR; OPV

^a^BCG: Bacillus Calmette–Guérin.

^b^DTaP-Hib-IPV + HBV: diphtheria, tetanus, pertussis (whooping cough), polio, *Haemophilus influenzae* type b, and hepatitis B.

^c^RV: rotavirus vaccine.

^d^OPV: oral polio vaccine.

^e^MMR: measles, mumps, and rubella.

^f^DTP: diphtheria, tetanus, and pertussis.

^g^Td: tetanus and diphtheria.

### Children Immunization App Design and Security Clearance at Zaatari Camp

During the period from August to January 2019, the CImA app has been designed, developed in English and Arabic languages [26], and tested in house. In parallel to the app design and development, we have sought and obtained the security clearance from the office of the UNHCR, which has the full mandate of protecting the Zaatari camp.

### Assessments

Baseline will include the collection of our primary and secondary outcomes that are needed for the pre and postdata measurements. This includes social demographic data, any previous vaccination history, and electronic health literacy [27]. Participants, in both study arms, will be monitored for their follow-up visits to the clinic for the vaccination doses. The vaccination cards of both study arms will be marked as *intervention* or *control* arm; thus, the clinic nurses will notify the field workers about the follow-up visits. For the study outcome measures, we will measure any differences in the proportion coming back on time for the follow-up vaccination visit. The secondary outcome is the utility of the CImA app.

### Statistical Analysis

The analysis will be done using a set of steps. The baseline characteristics of the participants will be compared between the intervention and control groups, using independent 2-tailed *t* test for continuous variables and Chi-square test for categorical variables. Delta analysis (D%) will be used to assess the change in the vaccination rate between preintervention (t0) and postintervention (t1), contrasting this change within the intervention and control groups. This will be complemented by an effect size assessment to quantify the difference in change between the 2 groups (Cohen *d* test will be used). Hierarchical level modeling will be conducted to consider any cluster effect of the clinics (defaulters will be treated as intent-to-treat analysis in the primary outcome of the study). For the secondary outcome, survival analysis will be used to further contrast the difference in the secondary outcome (proportion of defaulters) between the intervention and comparison groups, postintervention (t1). Stata will be used for the data analysis.

### Ethical Considerations

This study has been reviewed and approved by the Institutional Review Board of the Jordan University of Science and Technology (Reference# 14/112/2017, date January 14, 2018). In addition, the project proposal has been endorsed by the Minister of Health in Jordan, UNICEF-Jordan. Owing to the vulnerability of the refugees and the context of the camp, all participants will be invited to participate on a volunteer basis. Survey data will be collected at baseline followed by a survey at the end of the study. Participants will be given ID numbers, and these numbers will be stored in the app of each participant. No personal information will be stored. The research assistants will review the schedule of the app and match it against the standardized tool for the vaccination schedule of Jordan-Ministry of Health. Study participants will have their full right to cancel their participation in the study, including closing their study file. The data will be entered and stored in a secured database, using EpiData [28], and only team members will have access to it. Any reports or publications will be anonymized, and no identifying information will be included.

### Benefits for the Refugees

Few studies exist in the published literature, which focus on vaccination, health information, and the needs of refugee population [29,30]. This study will determine the immunization coverage gap and attempt to reduce it among refugee population in 3 different settings and evaluate a newly designed and piloted app to replace traditional paper-based immunization and health care records, which are not an option among a traveling/mobile population [31]. This will enable refugees to keep a record of vaccinations and prevent VPDs.

### Communications and Dissemination Strategy

Before the launching of the study in March 2019, we held a briefing meeting with the representatives of the clinics, social workers, and health care workers of the camp. Thereafter, bimonthly progress meetings will be conducted with the same group of representatives. This will help the development of the policy document related to immunization in the 3 countries. The results will be published in open-access peer-reviewed international scientific journals to enable wider access inside and outside these countries, especially among low- and middle-income contexts. This will benefit other countries in improving their vaccination programs, using smartphones.

## Results

The study enrolment is still ongoing. We expect to complete the study by October 2019 followed by data analysis and submission of the final report by the end of 2019.

## Discussion

This paper describes the study protocol for a randomized controlled trial of a smartphone app on child vaccination at one of the largest Syrian refugee camps in the world. Owing to the current gap in children vaccination coverage in Jordan, this study will provide an important preliminary insight on using a smartphone app to improve child immunization in a refugee camp, and this study will be unique in certain ways. First, according to our knowledge, this is the first cluster randomized controlled trial of a smartphone app–based intervention to support the national vaccination program. This includes the effects on the immunization coverage, follow-up visits, and the experience of both of health care staff and parents in using the app. The outcome of this study will be of high importance for the different collaborators, including UNICEF, UNHCR, and the Jordan-Ministry of Health.

### Conclusions

Owing to the limited evidence of effective interventions for childhood vaccination among refugees, research in this area is greatly needed. The project will have a significant impact on the health of refugees and the public health system. In Jordan, and the Middle East, the vaccination level is low. Given the influx of refugees from the area, it is crucial to ensure a high vaccination level for the refugees, especially for children, to avoid VPDs.

## Appendix

Multimedia Appendix 1 Peer-review reports from Grand Challenges Canada.

## Abbreviations

CImA: children immunization app
mHealth: mobile health
UNHCR: United Nations High Commissioner for Refugees
UNICEF: United Nations Children’s Fund
VPD: Vaccine Preventable Disease
