# Supplementary material for: Children Immunization App (CImA) Among Syrian Refugees in Zaatari Camp, Jordan: Protocol for a Cluster Randomized Controlled Pilot Trial Intervention Study
Source: JMIR Res Protoc. 2019 Oct 7;8(10):e13557. doi: 10.2196/13557 (PMC6803890; doi:10.2196/13557)
Supplement: Multimedia Appendix 1 [file resprot_v8i10e13557_app1.docx]

**Canadian Institute of Health Research
Reviewer's Comments:**

**Reviewer 2:**

The proposal has the potential for an important impact – serving displaced/migrant communities – Syrian refugees as they migrate. The proposal was confusing in that it addressed the development of the app and translation into Arabic. But, it seems like this has already been completed. It seems that this proposal is either about performing a trial or implementation – but the proposal was not clear.

It would have been helpful if that proposal had addressed the cellphone penetration of the Syrian refugee population as a way of quantifying the potential for impact with this solution.

Good video. Seemed more thoughtful and focused than the written application. Integrated innovation was not addressed in the proposal.

This is not about scaling – it is about testing or implementing an intervention. The proposal did not address the potential for scaling the Arabic language version of CANImmunize. The team is well-assembled.

The proposal did not address the what happens when people migrate – and whether they will be lost to follow up.

Some articulation about the transit time through the camps would have been helpful. This project has great potential for populations beyond the current study/implementation population (Syrian refugees).

**Reviewer 1:** The disrupted vaccination schedule of refugee children in Syria is an urgent healthcare issue that need to be addressed. The proposed method of storing vaccination record in a cell phone and sending reminders for upcoming vaccination sounds useful to improve the timeliness of vaccination in refugee children. However, more evidence is need to demonstrate this ‘digital yellow card’ will facilitate the timely vaccination of refugee children as there may be other technical and social barriers that interfere with the vaccination schedule. The technical innovation is not high as what is proposed is the recording of immunization and reminder of the upcoming ones by cell phone. Social and business innovations are not discussed much. The scaling-up of the mobile app will mainly rely on collaboration with the country’s ministry of health. The team includes several investigators tor cover different aspects of this project. The technical details of the study is outlined. A 12-month timeline is provided, and the breakdown the fund spending does not indicate the purpose of the spending. The transformative perspective of this project is relatively high.

**Reviewer 3:** The proposal could significantly improve the data accuracy on child vaccination in refugee camps, reduce the cost and effectiveness vaccination programs, increase vaccination rates and improve newborn and child health. The proposal is targeting refugee families with children, especially those who have smart phones. The project partners are carefully selected – MOH, UNICEF, UNHCR and academic partners for successful implementation and potential scale up. It seems that the initial consultation with the government, NGO and academic partners raised significant interest and support. It looks like the proposed application has been tested and approved in other settings. There seems to be potential for successful implementation of the Arabic version of the app and for testing the concept in refugee camps in Jordan and other countries in the region accepting Syrian refugees. The project team proves to be a experienced in mHealth and similar projects. The study design to test the implementation on the innovation is plausible. With high uptake of the proposed app, the project has a potential to significantly improve the vaccination follow ups and coverage.

**PO Comments:** Please address comments/questions/concerns from Reviewer 2.
